# The Influence of Proton Pump Inhibitors on the Fecal Microbiome of Infants with Gastroesophageal Reflux—A Prospective Longitudinal Interventional Study

**DOI:** 10.3389/fcimb.2017.00444

**Published:** 2017-10-11

**Authors:** Christoph Castellani, Georg Singer, Karl Kashofer, Andrea Huber-Zeyringer, Christina Flucher, Margarita Kaiser, Holger Till

**Affiliations:** ^1^Department of Paediatric and Adolescent Surgery, Medical University of Graz, Graz, Austria; ^2^Institute of Pathology, Medical University of Graz, Graz, Austria

**Keywords:** proton pump inhibitors, microbiome, infants, GERD, *Clostridium difficile*

## Abstract

Proton pump inhibitors (PPIs) are the standard therapy for gastroesophageal reflux disease. In adults, PPI treatment is associated with *Clostridium difficile* infections (CDI). In contrast to adults the microbiome of infants develops from sterility at birth toward an adult-like profile in the first years of life. The effect of PPIs on this developing microbiome has never been studied. The aim of the present study was to determine the effect of oral PPIs on the fecal microbiome in infants with gastroesophageal reflux disease (GERD). In this prospective longitudinal study 12 infants with proven GERD received oral PPIs for a mean period of 18 weeks (range 8–44). Stool samples were collected before (“before PPI”) and 4 weeks after initiation of PPI therapy (“on PPI”). A third sample was obtained 4 weeks after PPI discontinuation (“after PPI”). The fecal microbiome was determined by NGS based 16S rDNA sequencing. This trial was registered with clinicaltrials.gov (NCT02359604). In a comparison of “before PPI” and “on PPI” neither α- nor β-diversity changed significantly. On the genus level, however, the relative abundances showed a decrease of *Lactobacillus* and *Stenotrophomonas* and an increase of *Haemophilus*. After PPI therapy there was a significant increase of α- and β-diversity. Additionally, the relative abundances of the phyla Firmicutes, Bacteroidetes, and Proteobacteria were significantly changed and correlated to patients' age and the introduction of solid foods. PPI treatment has only minor effects on the fecal microbiome. After discontinuation of PPI treatment the fecal microbiome correlated to patients' age and nutrition.

## Introduction

Gastroesophageal reflux (GER) is a common finding in infants caused by temporary relaxations of the immature lower esophageal sphincter (LES) (Vandenplas et al., 2007). With maturation of the LES in the first year of life GER events often decrease. Some infants, however, may develop gastroesophageal reflux disease (GERD) associated with vomiting, feeding problems, pain, esophagitis, failure to thrive and/or recurrent respiratory infections (Rudolph et al., 2001; Colletti and Di Lorenzo, 2003).

In these infants conservative therapy includes upright positioning, increased feeding frequencies with lower amounts and food thickeners (Hollwarth, 2012). Nevertheless, some children may require acid suppression therapy with proton pump inhibitors (PPI). In adults, possible side effects of prolonged PPI therapy include an increased risk of community acquired enteritis and *Clostridium difficile* infections (CDI) (Janarthanan et al., 2012; Bouwknegt et al., 2014; McDonald et al., 2015).

The influence of PPI therapy on the intestinal microbiome has only been studied in adults under PPI therapy demonstrating dramatic changes of both the gastric and esophageal microbial communities (Amir et al., 2014). Furthermore, examinations of fecal samples have shown an increased abundance of *Enterococcae* and *Streptococcae* as well as decreased *Clostridiales*, associated with an increased risk of CDI (Freedberg et al., 2015b). Recent reports also describe an increased risk of CDI infections in infants under acid suppression treatment (Brown et al., 2015; Freedberg et al., 2015a). The exact pathophysiological mechanism of this association, however, is poorly understood. The two most common theories are (1) PPI directly affect the microbial environment by increasing the gastric pH and/or (2) PPI directly target bacterial proton pumps containing P-type ATPase enzymes (Vesper et al., 2009).

The gut microbiome in infancy develops from sterility at birth to an adult-like profile [dominated by the phyla Firmicutes (50–70% total bacterial numbers), Bacteroidetes (10–30%), Proteobacteria (up to 10%) and Actinobacteria (up to 10%), (Eckburg et al., 2005)] within the first years of life (Palmer et al., 2007; Yatsunenko et al., 2012). In this period a longitudinal investigation of fecal samples has revealed an increase of the total number of colonizing bacteria as well as unstable and heterogenic relative abundances of the different phyla (Palmer et al., 2007). Thus, data derived from the “stable” microbiome in adults are not representative for infants (Palmer et al., 2007; Yatsunenko et al., 2012).

PPI-associated changes of the microbiome have not been studied in infancy yet. Therefore, the aim of this prospective longitudinal interventional investigation was to assess the influence of oral PPI therapy on the fecal microbiome of infants with proven GERD.

## Materials and methods

According to our institutional protocol all patients with suspected GERD undergo 24 h-pH-impedance monitoring (24 h-pH-MII). After ethical approval (Ethical Committee of the Medical University of Graz, 26-429 ex 13/14) and informed consent of parents or legal guardians patients younger than 1 year with proven GERD were enrolled in this study between November 2014 and August 2016. Patients with relevant additional diagnoses were excluded. This trial was registered with clinicaltrials.gov (NCT02359604).

A first stool sample was taken before initiation of PPI therapy, stored in a PSP® Spin Stool DNA Kit (Stratec molecular GmbH, Berlin, Germany) and frozen at −21°C until further processing (“*before PPI”* sample). According to our protocol all patients received 1 mg/kg body weight oral esomeprazole daily. After 4 weeks of PPI treatment a second stool sample was collected and stored as described (“*on PPI”* sample). The duration of PPI therapy depended on the patients' clinical symptoms. A third sample was collected 4 weeks after discontinuation of PPI therapy (“*after PPI”* sample). None of the patients received antibiotics or other acid suppressants during the course of the study. Dietary habits were recorded.

### DNA isolation, 16s library preparation and sequencing

Frozen stool samples were thawed and a peanut sized stool sample was thoroughly mixed in 500 μl PBS. 250 μl of the suspension were mixed with 250 μl bacterial lysis buffer from the MagnaPure LC DNA Isolation Kit III (Bacteria, Fungi) (Roche, Mannheim, Germany) and transferred to MagnaLyser green bead tubes (Roche, Mannheim, Germany) for mechanical lysis performed two times at 6,500 rpm for 30 s in a MagnaLyser instrument (Roche, Mannheim, Germany). After bead beating 25 μl lysozyme (100 mg/ml) were added to the samples for enzymatic lysis and incubated at 37°C for 30 min followed by incubation with 43 μl Proteinase K (20 mg/ml) at 65°C for 1 h. Heat inactivation of enzymes was performed at 95°C for 10 min. Samples were centrifuged at 13,000 rpm for 5 min and 100 μl of the lysed samples were transferred to the Magna Pure instrument and DNA was purified according to manufacturer's instructions. PCR and library preparation with hypervariable regions V1-2 were performed as described before (Klymiuk et al., 2016) with 2 μl of total DNA per 25 μl PCR reaction in triplicates using primers 27f (AGAGTTTGATCCTGGCTCAG) and 357r (CTGCTGCCTYCCGTA) yielding a 330 bp long insert. Triplicates were pooled, amplification was verified by checking on a 1% agarose gel and sequencing library was normalized, indexed, and quantified according to Klymiuk et al. (2016). The pooled sample library was sequenced on a MiSeqII desktop sequencer (Illumina, Eindhoven, Netherlands) with v3 600 cycles chemistry (Illumina, Eindhoven, Netherlands) according to the manufacturer's instructions at 6 pM with 20% PhiX (Illumina, Eindhoven, Netherlands) in one run.

Sequence reads were submitted to the NCBI Sequence Read Archive (https://www.ncbi.nlm.nih.gov/sra/?term=SRP119055).

### Microbiome analysis

Sequencing reads were processed with scripts of the QIIME platform. Briefly, reads were clustered to Operational Taxonomic Units (OTU) using the pick_open_reference_otus.py script and uclust algorithm based on the greengenes database (gg_otus-13_8-release) and a 97% identity threshold. OTUs were visualized as OTU tables, bar charts and PCOA plots. Alpha diversity measurements (observed species and chao1) and beta-diversity measurements (unweighted unifrac) were derived using the respective QIIME tools. Group significance for all categories was determined with the Adonis test, while individual species difference was quantified by Kruskall-Wallis tests and pairwise comparisons by Mann-Whitney-U-test. The Adonis test computes R2 (effect size) and pseudo-P values of categories by first identifying the relevant centroids of the data and then calculating the squared deviations from these points. After that, significance tests are performed using *F*-tests based on sequential sums of squares from permutations of the raw data. Adonis tests were performed in R (2.15.1) using the vegan package. Significance of differences in alpha diversity was calculated by non-parametric two-sample *t*-test using Monte Carlo permutations to calculate the *p*-value. Lefse analysis was performed for all categories as described previously (Segata et al., 2011).

## Results

36 stool samples (*n* = 12 “before PPI”, *n* = 12 “on PPI,” *n* = 12 “after PPI”) of 12 patients (8 male, 4 female) were included. The mean gestational age was 38 weeks (STD 2.0; range 35–41 weeks). Patients had a mean birth weight of 2,794 g (STD 468; range 2,100–3,688 g) and a mean birth length of 48.8 cm (STD 2.6; range 44–53 cm).

Patients were included at a mean age of 5.2 months (STD 3.2; range 0.5–10.2 months). All patients suffered from GERD. The data of their 24 h-pH-MII is shown in Table 1. The patients' nutrition at the time of stool sampling is displayed in Table 2. The mean duration of PPI treatment was 18 weeks (STD 11; range 8–44).

**Table 1:** Results of the 24 h-pH-MII before initiation of oral PPI therapy (*n* = 12).

	**AET**	**ABET**	**WABET**	**NABET**	**TBET**	**NRA**	**NRWA**	**NRNA**	**NRT**
	**%**	**%**	**%**	**%**	**%**	***n***	***n***	***n***	***n***
Mean	7.3	1.0	0.8	0	1.8	34.3	25.4	0.4	60.0
STD	4.9	0.8	0.6	0	0.9	21.4	19.9	1.2	21.1
MIN	0.6	0.2	0.1	0	0.4	9.0	8.0	0.0	30.0
MAX	16.3	2.9	2.0	0.1	3.5	73.0	75.0	4.0	96.0

*AET, acid exposure time (pH < 4); ABET, acidic bolus exposure time (pH < 4); WABET, weakly acidic bolus exposure time (4 ≤ pH < 7); NABET, non-acidic bolus exposure time (pH ≥ 7); TBET, total bolus exposure time; NRA, number of acidic refluxes; NRWA, number of weakly acidic refluxes; NRNA, number of non-acidic refluxes; NRT, total number of refluxes*.

**Table 2:** Nutrition of the infants at the three different time points of stool sampling (*n* = 12).

**ID**	**1st sample**	**2nd sample**	**3rd sample**
	**Before PPI**	**On PPI**	**After PPI**
1	MM	MM	MM
2	FM	FM	FM/SF
3	MM/FM	FM	FM/SF
4	MM/FM	FM	FM/SF
5	FM	FM	FM/SF
6	FM	FM	FM/SF
7	FM	FM/SF	FM/SF
8	MM/SF	FM/SF	FM/SF
9	MM/FM	FM/SF	FM/SF
10	FM	FM/SF	FM/SF
11	MM/SF	FM/SF	FM/SF
12	MM/SF	MM/SF	FM/SF

*MM, mother's milk; FM, formula; SF, solid food*.

### PPI therapy had no influence on α- and β-diversity

**Figure 1:**
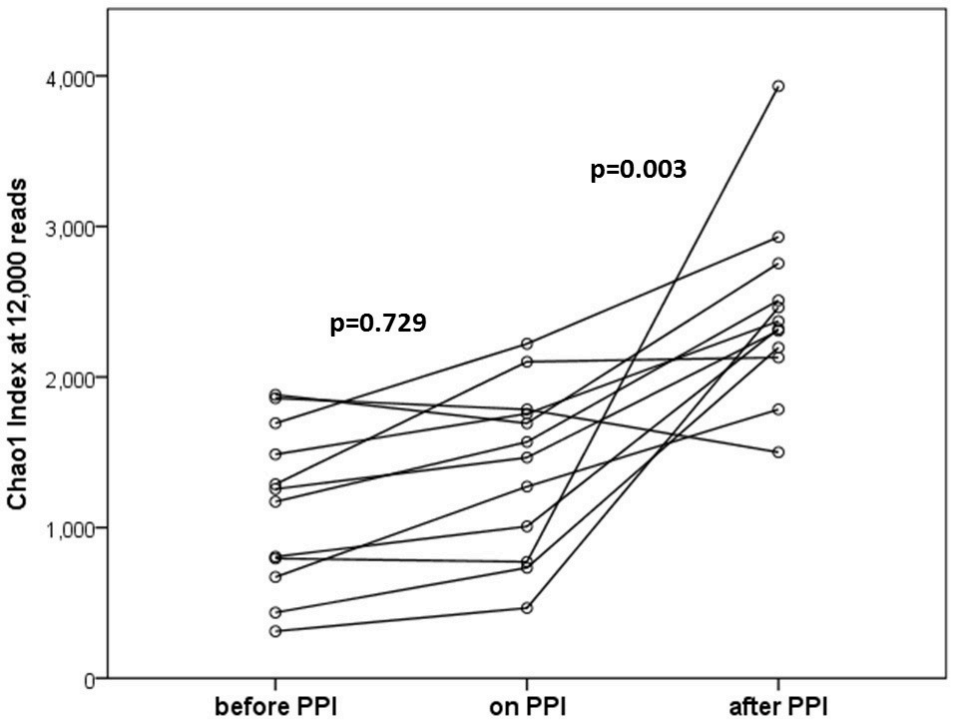
α-diversities (Chao1 index at 12,000 reads) at the three time points tested (*n* = 12 per time point). In the within-individual comparison there was no statistically significant difference after 4 weeks of PPI treatment (*p* = 0.729). Four weeks after discontinuation of PPI therapy α-diversities were significantly increased (*p* = 0.003 for “after PPI” vs. “before PPI” and “on PPI.” Lines connect individuals.

In the within-individual comparison (“before PPI” vs. “on PPI”), oral PPI treatment did not influence α-diversity (Chao1 index; *p* = 0.729, Figure 1). Additionally, β-diversity did not change when comparing “*before PPI*” and “*on PPI*” (unweighted UniFrac; *p* = 0.913).

### PPI treatment caused only minimal changes in the fecal microbiome

**Figure 2:**
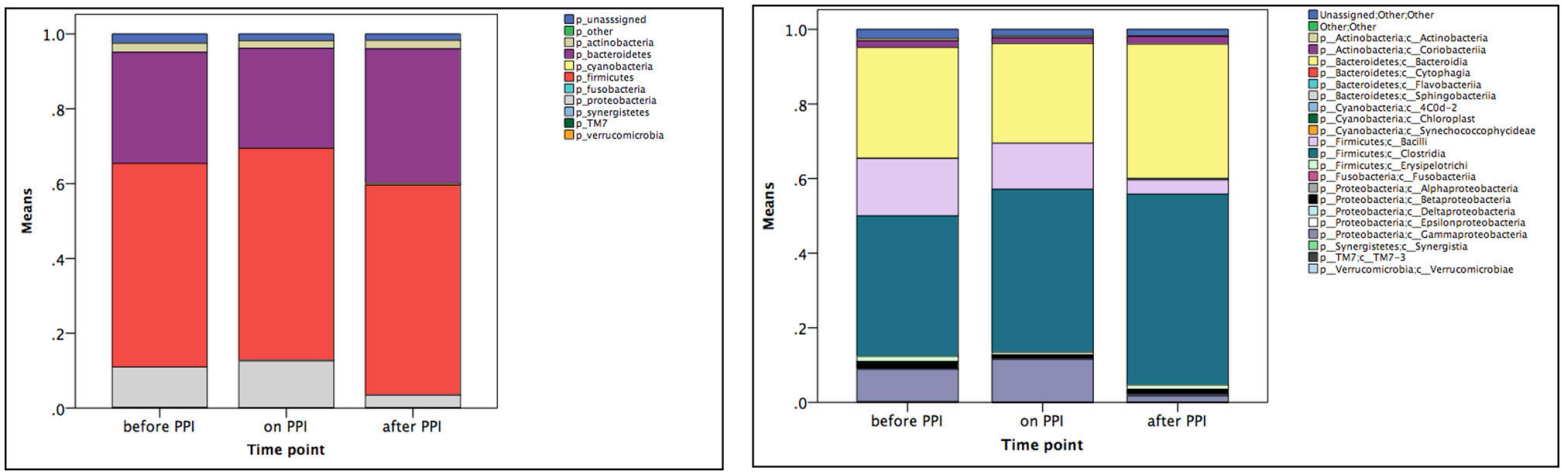
Mean taxa summary plots at the phylum (left panel) and class (right panel) level at the three different time points measured (*n* = 12 per time point).

**Figure 3:**
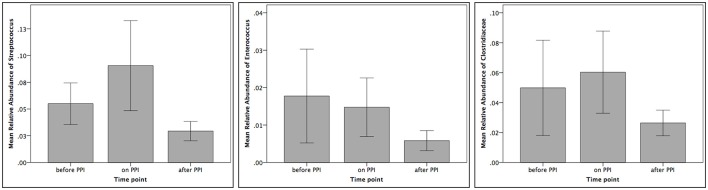
Relative abundances of *Streptococcus, Enterococcus* and *Clostridiaceae* at the three different time points tested (*n* = 12 per time point).

Taxa summary plots at the phylum and class level at the different time points tested are depicted in Figure 2. On the genus level, PPI therapy caused a significant decrease of *Lactobacillus* and of *Stenotrophomonas*. Additionally, there was a significant increase of *Haemophilus* (Table 3). Although Streptococcus increased under PPI therapy none of the bacteria associated with an elevated risk of CDI in adults (*Streptococcus, Enterococcus, Clostridiaceae*) were significantly altered (Figure 3). There was no significant correlation between the results of impedance testing and the corresponding microbiome (“*before PPI*”) (*p* > 0.10).

**Table 3:** Mean relative abundances (RA) at the different levels.

**Phylum**	**Class**	**Order**	**Family**	**Genus**	**Before PPI**	**On PPI**	**After PPI**
Firmicutes					0.544	0.565	0.551
	*Clostridia*	*Clostridiales*	*Ruminococcaceae*		0.040	0.044	0.095^*^^#^
				*Oscillospira*	3.1 × 10^−3^	1.7 × 10^−3^	6.4 × 10^−3^^*^^#^
				*Faecalibacterium*	3.3 × 10^−3^	3.7 × 10^−3^	9.2 × 10^−3^
				*Ruminococcus*	2.9 × 10^−3^	2.1 × 10^−3^	5.1 × 10^−3^^*^^#^
			*Veillonellaceae*		0.128	0.107	0.056^*^^#^
				*Veilonella*	0.119	0.096	0.026^*^^#^
				*Phascolarctobacterium*	0	3.7 × 10^−7^	9.3 × 10^−3^^*^
			*Lachnospiraceae*		0.115	0.158	0.249^*^
				*Lachnospira*	4.3 × 10^−3^	0.016	0.018^*^^#^
				*Blautia*	0.015	0.017	0.036^*^^#^
				*Coprococcus*	6.8 × 10^−3^	7.5 × 10^−3^	0.022^*^^#^
				*Dorea*	4.8 × 10^−4^	1.3 × 10^−4^	1.3 × 10^−3^^*^^#^
				*Anaerostiges*	7.2 × 10^−5^	0	0
	*Bacilli*	*Lactobacillales*	*Lactobacillaceae*	*Lactobacillus*	2.6 × 10^−4^	2.9 × 10^−5^^*^	3.4 × 10^−5^^*^
	*Erysipelotrichi*	*Erysipelotrichales*	*Erysipelotrichaceae*	*Eubacterium*	2.6 × 10^−3^	5.5 × 10^−4^	3.7 × 10^−3^^*^^#^
				*Coprobacillus*	3.2 × 10^−5^	1.2 × 10^−4^	1.8 × 10^−3^^*^^#^
Bacteroidetes					0.302	0.272	0.376
	*Bacteroidea*	*Bacteroidales*	*Rikenellaceae*	*Bacteroides*	6.3 × 10^−5^	4.8 × 10^−5^	0.010^#^
Proteobacteria					0.11	0.13	0.03^*^
	*Gammaproteobacteria*				0.087	0.117	0.017^*^^#^
		*Enterobacteriales*	*Enterobacteriaceae*	*Escherichia*	0.086	0.110	0.017^*^^#^
		*Xanthomonadales*	*Xanthomonadaceae*	*Stenotrophomonas*	5.1 × 10^−5^	1.6 × 10^−6^^*^	1.6 × 10^−6^^*^^#^
		*Pasteurellales*	*Pasteurellaceae*	*Haemophilus*	0	2.3 × 10^−5^^*^	1.8 × 10^−6^
Acinetobacteria					0.024	0.020	0.023
	*Coriobacteria*	*Coriobacteriales*	*Coriobacteriaceae*	*Adlercreutzia*	0	0	3.7 × 10^−5^
Cyanobacteria					2.2 × 10^−5^	3.1 × 10^−6^	3.8 × 10^−3^
Verrucomicrobia					1.7 × 10^−3^	0	4.9 × 10^−4^
Fusobacteria					5.4 × 10^−4^	1.5 × 10^−3^	4.1 × 10^−4^
TM7					2.3 × 10^−5^	4.5 × 10^−5^	7.0 × 10^−5^

* p < 0.05 vs. “before PPI”;

# *p < 0.05 vs*.

*“on PPI.” Significant increases under PPI therapy are colored in blue, significant decreases in red*.

### The third sample (“after PPI”) showed increasing α- und β-diversities and altered relative abundances in correlation with patients' age and dietary habits

The α-diversity significantly increased over the time of the experiment (*p* = 0.003 for the comparison “*before PPI*” to “*after PPI*” and *p* = 0.003 for the comparison “*on PPI*” and “*after PPI*”). Furthermore, the β-diversity significantly changed throughout the experiment (*p* = 0.003 comparing “*before PPI*” and “*after PPI*” and *p* = 0.001 comparing “*on PPI*” and “*after PPI*”). For the relative abundances the majority of changes was also seen in comparison to the third sample and occurred in the Firmicutes phylum (Table 3). A correlation was found between these microbial changes and the patients' age (*p* = 0.062) and nutrition (*p* = 0.001).

## Discussion

This study is the first to address fecal microbial changes under PPI treatment in infants. In contrast to most studies reported in adults GERD was not only suspected in our patients, but proven by impedance monitoring prior to enrollment and PPI therapy. Notably, we were able to find a completely different response to PPIs in infants than previously described in adults.

Up to now the majority of studies investigating the effect of PPI on gut microbiota have compared adult PPI users to non-users. Two large series have reported significant decreases of the overall fecal microbial diversity under PPI treatment (Imhann et al., 2016; Jackson et al., 2016). However, to assess the exact effect of PPIs on individuals a longitudinal investigation of the same patients on and off PPI is required. Presently there is only one study in 12 adults which has addressed this issue reporting no significant changes of the fecal microbial diversity (Freedberg et al., 2015b). Similarly, our investigation with the same sample size in infants showed no significant changes of α- and β-diversity under PPI treatment.

Regarding relative bacterial abundances under PPI several reports with varying findings in adult patients have been published. Overall, *Streptococcus, Enterococcus*, and *Clostridiales* were most commonly affected in this population. Imhan et al., for instance, have described significant increases of *Enterococcus, Streptococcus*, and *E. coli* under PPI therapy (Imhann et al., 2016). Another group has found increased abundances of *Streptococcaceae, Lactobacillaceae, Pasteurellaceae, Corynebacteriaceae*, and *Micrococcaceae* (amongst others) and decreases of *Lachnospiraceae, Ruminococcaceae*, and *Erysipelotrichaceae (Jackson et al., 2016)*. Furthermore, an observation of fecal samples of long-term PPI users has revealed an increase of *Lachnospiraceae, Erysipelotrichaceae*, and *Streptococcaceae (Clooney et al., 2016)*. In contrast to adults our investigation in infants revealed only minor changes of the relative microbial abundances under PPI therapy.

In adults, an increased risk of *Clostridium difficile* infections (CDI) under PPI therapy has been postulated. In detail, increases of *Enterococcae* and *Streptococcae* combined with decreases of *Clostridiales* were reported in association with an increased risk of CDI (Freedberg et al., 2015b). Similarly, recent pediatric studies have reported an increased risk of CDI under acid suppression therapy (Brown et al., 2015). First reports about associations between PPI and CDI infections in infants (Freedberg et al., 2015a) rely on culture-based retrospective investigations only. In our series we have found a non-significant increase of *Streptococcus* and *Clostridiaceae* under PPI treatment (compare Figure 3). These results further fuel recent controversial discussions regarding the association between CDI and PPI (Leffler and Lamont, 2015; Faleck et al., 2016).

The majority of changes in our series were seen when comparing the microbiome between “*before PPI*” and “*on PPI*” to “*after PPI*.” While we cannot rule out the possibility that the removal of PPI treatment may cause a temporary flux in diversity, other studies showing an increasing diversity with increasing infants' age (Hill et al., 2017) support the physiological development of the intestinal microbiome as the underlying reason for this finding. Additionally, the correlation between the relative abundances and patients' age/nutrition also suggests the developing microbiome of infants as the most likely cause for these alterations.

One possible limitation of the present study includes the lack of a control group without PPI treatment. However, recent investigations of the developing microbiome have shown a marked variability and heterogeneity of the fecal microbiome within the first years of life (Palmer et al., 2007; Yatsunenko et al., 2012). This makes the selection of infants for a representative and comparable control group difficult. In accordance to the literature we have found a constant increase of α-diversity in our samples (Palmer et al., 2007). This further substantiates the developing microbiome as a reason for the changes encountered in the third sample of this series. In our longitudinal intra-individual comparison, the same patient is investigated on and off PPI and thus serves as his/her own control. Another limitation is the relatively small number of included infants. However, the sample size resembles that of the only longitudinal study in adult patients (Freedberg et al., 2015b). Our setting also takes care of the heterogeneity of the microbiome in infants and the different age of our patients upon inclusion because the patients are compared by dependent tests within themselves (intra-individual).

Finally, we have only measured the fecal microbiome and could not include samples from other parts of the gastrointestinal (GI) tract. The fecal microbiome is easily accessible and the obtained results can be compared to adult studies (Freedberg et al., 2015b; Clooney et al., 2016; Imhann et al., 2016; Jackson et al., 2016). However, the fecal microbiome does not necessarily represent the whole GI-tract (Haange et al., 2012) and possible alterations of the microbial diversity of the upper GI-tract under PPI treatment are subject for further investigations.

Since we were not able to demonstrate any relevant changes under PPI therapy one might question whether a PPI dosage of 1 mg/kg/day esomeprazole was sufficient. Theoretically, our findings could be caused by inadequate acid suppression. Although we did not repeat impedance monitoring “*on PPI*” we could demonstrate that our institutional protocol and PPI dosage was effective in a previous examination (Castellani et al., 2014). Additionally, the symptoms caused by the GERD resolved in all our patients under PPI therapy suggesting adequate acid suppression.

In conclusion, oral PPI therapy did not have relevant impact on the development of the infant fecal microbiome at a sensitive time of life in our series. Microbial changes associated with an increased risk of CDI infection described in adults did not reach statistical significance in this study. The majority of alterations occurred through the course of time and is correlated to patients' age and nutrition representing the normal development of the microbiome. Future studies are required to investigate possible microbial changes of the upper GI-tract under PPI treatment.

## Author contributions

CC and GS performed the statistics and wrote the manuscript; KK performed the microbiome analysis and performed the biostatistics; AH recruited the patients and collected the samples; CF and MK analyzed the data and assisted to draft the manuscript; HT coordinated the project and critically reviewed the manuscript.

### Conflict of interest statement

The authors declare that the research was conducted in the absence of any commercial or financial relationships that could be construed as a potential conflict of interest.
